# Persistence of Marine Bacterial Plasmid in the House Fly (*Musca domestica*): Marine-Derived Antimicrobial Resistance Genes Have a Chance of Invading the Human Environment

**DOI:** 10.1007/s00248-023-02341-4

**Published:** 2024-01-08

**Authors:** Kanoko Nawata, Aya Kadoya, Satoru Suzuki

**Affiliations:** 1 Center for Marine Environmental Studies, Ehime University, Matsuyama, Ehime, Japan; 2 Graduate School of Science and Engineering, Ehime University, Matsuyama, Ehime, Japan

**Keywords:** House fly, Marine bacteria, Plasmid, Transconjugant, Antibiotic resistance gene, Excrement

## Abstract

The house fly is known to be a vector of antibiotic-resistant bacteria (ARB) in animal farms. It is also possible that the house fly contributes to the spread of ARB and antibiotic resistance genes (ARGs) among various environments. We hypothesized that ARB and ARGs present in marine fish and fishery food may gain access to humans via the house fly. We show herein that pAQU1, a marine bacterial ARG-bearing plasmid, persists in the house fly intestine for 5 days after fly ingestion of marine bacteria. In the case of *Escherichia coli* bearing the same plasmid, the persistence period exceeded 7 days. This interval is sufficient for transmission to human environments, meaning that the house fly is capable of serving as a vector of marine-derived ARGs. Time course monitoring of the house fly intestinal microflora showed that the initial microflora was occupied abundantly with Enterobacteriaceae. Experimentally ingested bacteria dominated the intestinal environment immediately following ingestion; however, after 72 h, the intestinal microflora recovered to resemble that observed at baseline, when diverse genera of Enterobacteriaceae were seen. Given that pAQU1 in marine bacteria and *E*. *coli* were detected in fly excrement (defined here as any combination of feces and regurgitated material) at 7 days post-bacterial ingestion, we hypothesize that the house fly may serve as a vector for transmission of ARGs from marine items and fish to humans via contamination with fly excrement.

**Supplementary Information:**

The online version contains supplementary material available at 10.1007/s00248-023-02341-4.

## Introduction

Antibiotics and synthetic antimicrobials are widely used for the control of infectious diseases in humans and animals [1] and as growth promoters in poultry and pigs [2]. However, the use for growth promotion has been banned in many countries, although antimicrobials continue to be released into the environment. Exposure of bacteria to such drugs creates selective pressure leading to the development of antibiotic-resistant bacteria (ARB). Furthermore, dissemination of antibiotic resistance genes (ARGs) is a matter of concern, not only in clinical settings but also in the environment. This challenge has led to the One Health concept [3], which postulates a strong connection between the health of humans and animals in an environmental context. ARGs released into environment can be considered genetic contaminants that circulate throughout various environments [4]. The connection between human and animal environments is the subject of ongoing attention; for instance, these environments have been shown to share vectors and carriers for ARGs [5].

One of the common vectors between humans and animals are insects. Antibiotic-resistant enterococci and staphylococci have been isolated from flies in poultry operations [6], and the transmission of ARB between cattle barns [7] and swine farms [8] has been reported. In those studies, clonal ARGs were detected in flies and animal feces, suggesting that flies are transmission vectors in farms. Since insects (including flies and cockroaches) typically are present in food-handling facilities, these organisms are expected to serve as vectors for the transmission of ARGs to humans [5]. Indeed, when the habitats of humans, animals, and flies overlap, the ARB carried by the flies often share the genotypes of bacteria in humans and animals [9]. Transmission by insect vectors likely is highest for enteric bacteria, given that such microbes are shed in high concentration in human and animal feces, a matrix that subsequently is ingested by flies. Flies fed on feces are expected to exhibit an increased risk of the transmission of ARGs as a result of bacterial growth and horizontal gene transfer (HGT) in the insect digestive tract.

A role of the house fly in ARG transmission also has been reported in wastewater treatment plants, where the sludge to be processed typically contains ARB [10]. This observation suggests that the house fly can serve as a vector between humans and water environments, just as these insects serve as a vector between humans and animals. ARGs are known to be present not only wastewater but also in seawater, including marine aquaculture sites [11–13] and the open ocean [14]. As an example of the abundance of ARGs in seawater, the tetracycline resistance gene *tet*(M) was detected in coastal seawater away from aquaculture sites at a mean level (over the course of a year) of 10^−5^ copies per 16S rRNA copy [15]. Tetracyclines have been used frequently worldwide in aquaculture [16], directly resulting in selective pressure leading to the development of ARB and ARGs in aquaculture settings and fish. The *tet* genes have been reported to be both abundant and persistent in the coastal environment [12, 17]. The prevalent ARGs (e.g., *tet* genes) are suspected to circulate between marine and human environments, with fresh fish and processed foods serving as point sources [18]. Given that fish markets typically harbor flies, these insects are hypothesized to gain contact with ARB (and ARGs) via fish in this context. Previous work has shown that the aquatic bacterium *Aeromonas hydrophila* survives for 24 h following ingestion by the house fly, although the majority of the cells of this species are lysed by 8 h [19]. ARB are ephemeral residents of house flies until the bacteria are excreted (by either defecation or regurgitation), resulting in the contamination of foods and/or goods used by humans.

Together, these results suggest that ARB survive, and ARGs persist, in flies for a period sufficient to permit the transmission of ARGs between different environments. However, the idea that house flies can act as a source of marine-derived ARB or ARGs has not (to our knowledge) been confirmed experimentally. We here aim to reveal the potential risk of flies for ARGs invasion to human environment from marine environment. Therefore, we examined whether house flies are a reservoir for a marine bacterial plasmid. The results presented herein demonstrate that house flies can serve as vectors for a marine bacterial plasmid, suggesting that these insects may facilitate the dissemination of ARGs between marine and terrestrial environments.

## Materials and Methods

### Rearing of House Flies

House fly (*Musca domestica*) pupae were purchased from the Sumika Technoservice Corporation (Takarazuka, Japan). Pupae were incubated at room temperature in plastic cages covered with polyester netting until emergence. Emerged adults were provided with sterile water and skim milk (Megmilk Snow Brand Co., Sapporo, Japan) dissolved in sterile water; the skim milk was supplemented with bacteria as described below. Both the water and milk sources were replaced daily. Individual experimental groups were maintained in cages separated by cardboard to avoid intergroup contamination.

### Experimental Groups and Sampling

Ingestion of bacteria was assessed using two species: the marine bacterium *Photobacterium damselae* subsp. *damselae* Strain 04Ya311, which harbors the pAQU1 plasmid [20], and a pAQU1-containing *Escherichia coli* strain, designated TJ-W3110, that was obtained by transconjugation of Strain W3110 with *P. damselae* Strain 04Ya311 [21]. In both *P. damselae* and *E*. *coli*, pAQU1 is a single-copy plasmid [22]. Bacteria were cultured by incubation at 25 °C (04Ya311) or 37 °C (TJ-W3110) with shaking (120 rpm) in LB broth (Becton Dickinson, Franklin Lakes, NJ) supplemented with 60 µg/mL of oxytetracycline (OTC, Nacalai Tesque, Kyoto, Japan). Following overnight growth, bacterial cells were collected by centrifugation (4000 × *g*, 10 min), then resuspended in 1 mL of phosphate-buffered saline (PBS). Since the cell numbers of these bacterial suspensions were measured in colony forming units (CFUs), the cell numbers were defined after starting experiment. The suspensions diluted in skim milk were not quite same, which were 1.3 × 10^9^ or 6.2 × 10^8^ CFU/mL; as a control, an equivalent volume of PBS was added to skim milk. The experiment consisted of three groups, each of which was provided with 4 h of ad libitum access to 5 mL skim milk supplemented with 04Ya311m (150 flies), TJ-W3110 (150 flies), or PBS (170 flies). Before and after feeding with bacteria- or PBS-supplemented skim milk (the pulse), all groups were provided with ad libitum access to skim milk neat (the chase). Using an insect suction tube, house flies (*n* = 8 or 10/time point/group) were collected just before the start of bacterial feeding (nominal “0 h”) and at 4, 8, 24, 72, 120, and 168 h after the start of bacterial feeding. Individual animals were washed once with 70% ethanol, then twice with PBS. Pairs of flies (from a given group) were pooled (placed in the same tube), thereby constituting a single specimen, meaning that there were 4–5 specimens/time point/group. Sampling scheme is shown in Fig. S1. Each tube was frozen and stored at − 25 °C until the time of DNA extraction. At the same times as the 0- and 168-h specimen collection, fly excrement (4 or 5 samples/time point/group) was collected from each cage by scraping with a sterile cotton swab. Note that this sampling approach did not distinguish between feces and regurgitated material; for clarity, therefore, these specimens are collectively referred to as excrement.

### DNA Extraction

DNA was extracted from the intestines and excrement of house flies with a NucleoSpin DNA Stool kit (Macherey–Nagel, Düren, Germany). To obtain the intestines, the abdomens of the flies in each specimen were separated from the body and placed in a tube containing 300 µL of Buffer ST1 containing 50 mM ethylenediaminetetraacetic acid (EDTA); the tube contents then were homogenized 5–6 times with a pestle. The resulting homogenate was added to NucleoSpin Bead tube type A, yielding a total volume of 940 µL. This mixture was incubated at 70 °C for 5 min, vortexed at maximum speed for 10 min, and centrifuged (13,000 × *g*, 3 min, room temperature). DNA was purified from the resulting supernatant by sequential use of Buffers ST2 to ST5 according to the manufacturer’s protocol. The final DNA fraction was stored at − 25 °C pending analysis. For DNA extraction from excrement, the cotton swab was immersed in a solution of 945 µL of ST1 + EDTA; the mixture was incubated at 56 °C for 5 min, then vortexed at maximum speed for 10 min. The resulting homogenate was subjected to centrifugation and DNA purification as for the intestinal samples above.

### Quantitative Analysis of Plasmid Copy Number

pAQU1 possesses 235 predicted coding sequences (CDSs) [20], including seven ARGs (*tet*(M), *tet*(B), *bla*_CARB_, *sul2*, *floR*, *mef*(C), and *mph*(G)) and the *tra* conjugative transfer genes. We employed one of the conjugative transfer genes, *traI*, for quantification of the plasmid copy number. This CDS is present as a single-copy gene on the plasmid. Copy number was quantified by polymerase chain reaction (PCR) using the primer pair *traI* F-2 (5′-AGAGGTAGTAGCTTCCCAGGTTAGG-3′) and *traI* R-2 (5′-GGCATGACTAAACGGTCGTACTCT-3′) [21]. PCR was performed using a program consisting of an initial denaturation at 95 °C for 30 s, followed by 40 cycles at 95 °C for 5 s and 50 °C for 10 s. Copy number was normalized to 16S rRNA gene copy number, which was quantified by PCR using the primer pair Bact1369F (5′-CGGTGAATACGTTCYCGG-3′) and Bact1492R (5′-GGWTACCTTGTTACGACTT-3′) [23]; for this PCR, the program consisted of an initial denaturation at 94 °C for 30 s, followed by 40 cycles at 94 °C for 15 s and 59 °C for 20 s. All amplifications were performed as 20-µL reactions in mixtures consisting of each primer at 500 nM primer and 1 ng of template DNA in 1 × SsoFast™ EvaGreen SuperMix (BioRad, Hercules, CA). Quantitative PCR was performed triplicate using a CFX96TM Real-Time System (BioRad).

### Microflora Analysis by 16S rRNA Metagenome

Microflora metagenomic analysis of the contents of the fly intestine was based on the V3-V4 region of the 16S rRNA gene sequence. As shown in Fig. S1, four or five of the DNA samples obtained (from a given cage/group) for use in plasmid quantification were pooled as a single specimen and employed for PCR using the primer pair 341F (5′-CCTACGGGNGGCWGCAG-3′) and 805R (5′-GACTACHVGGGTATCTAATCC-3′) [24]. The resulting PCR products were purified, and library was prepared and analyzed at Hokkaido System Science Co. (Sapporo, Japan). Steps from index-PCR (adapter addition) to library denaturing were conducted according to the protocol for 16S Metagenomic Sequencing Library Preparation for the Illumina MiSeq system (Illumina Inc., San Diego, CA). Library was analyzed by MiSeq Next-Generation Sequencing (NGS). Paired sequence reads exceeding 2 × 10^5^ per sample were obtained. Sequencing data were preprocessed by removing adapter sequences, trimming of low-quality reads, and paired-read joining; the data then were cleaned (by removing sequences of less than 200 bases or that included homopolymers) and processed for population analysis by QIIME 2 (version 2019.4.0). Reads including the trailing part of N bases or for which the 50-base average quality score was less than 25 were removed from the sequences. Operational taxonomic units (OTUs) were determined based on a 97% similarity threshold. The phylogenetic assignment of each OTU was carried out using the Greengenes 16S rRNA gene database (version 13_8). The assignments of some major OTUs were confirmed by comparison with the nucleotide collection of the NCBI database (as of May 30, 2023).

### Statistics

For the quantification of *traI* copy number, homogeneity of the data was determined by *F*-test. Statistical significance was assessed using Student’s *t* test (for homogeneous data distribution) or Welch’s *t* test (for heteroscedastic data). Both *t* tests were performed for all cross-combinations of samples. All analyses were conducted as two-tailed tests. In all samples (*n* = 4 or 5), values of *p* less than 0.05 were considered statistically significant. The β-diversities of the time course profiles of microflora were compared by principal coordinate analysis (PCoA) based on Bray–Curtis distance. Sampling depth was 1500. All statistical analyses were performed using the corresponding functions in QIIME 2 (version 2021.4).

## Results and Discussion

### Plasmid Copy Number in House Fly Intestine and Excrement

**Fig. 1:**
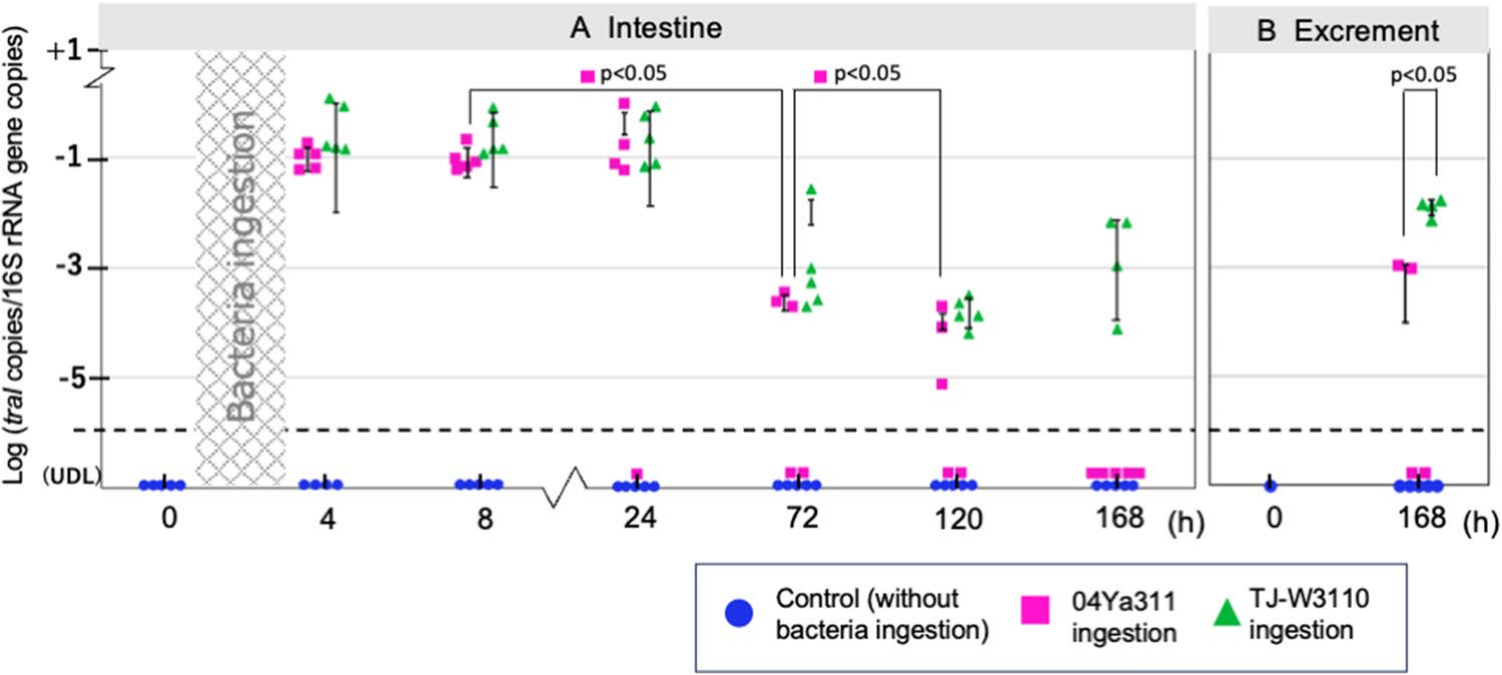
Time course of the abundance of the pAQU1 plasmid following 4 h of feeding on skim milk supplemented with vehicle (PBS) or bacteria. Plasmid levels are normalized to that of the 16S rRNA gene in the respective sample. A, house fly intestine; B, excrement (including feces and regurgitated material)

Plasmid copy numbers were quantified by analyzing (via PCR) the amount of the *traI* gene, as normalized to the copy number of the 16S rRNA gene; results are shown in Fig. 1. As shown in Fig. 1A, the control group (fed on milk not supplemented with bacteria) was negative for *traI* throughout the experimental time course. This observation was important: while pAQU1 originally was detected in marine bacteria [20, 25], a recent study detected pAQU1 in wastewater obtained from a pig farm in Taiwan [26], suggesting that this plasmid, while still not abundant, might be spreading in the environment. The present result confirmed that the house flies used in the present study do not carry this plasmid (as assessed by *traI*) as part of their native intestinal microbiome.

After 4 h of access to milk containing 04Ya311 or TJ-W3110, house fly intestines showed *traI* at high copy number until 24 h; these numbers subsequently declined at 72 h. Especially in the 04Ya311-fed group, plasmid number decreased by 72 h and decreased further by 120 h; all specimens of this group tested negative for the plasmid by 168 h. On the other hand, the TJ-W3110-fed group showed high copy number in all specimens, even at 120 and 168 h. These data indicated that the pAQU1 carried by TJ-W3110 (an *E. coli* transconjugant) was retained in the fly intestine for a longer interval than was 04Ya311 (a marine bacterium). Therefore, it appeared that the pAQU1 plasmid in 04Ya311 is degraded more rapidly (presumably concomitant with digestion of host cells) than that in TJ-W3110, suggesting that *E*. *coli* can survive or grow more effectively than *P*. *damselae* in the fly intestine. In case of excrement (which was expected to consist of both feces and regurgitated material), the pAQU1 copy number was higher for the flies fed TJ-W3110 than for those fed 04Ya311 (Fig. 1B**)**, supporting the longer persistence of the plasmid in ingested *E*. *coli* than in the ingested marine bacterium.

Notably, the present study showed that a marine bacterial plasmid was retained in house flies for at least 5 days, an interval that is expected to be sufficient for transmission to humans, even for *P*. *damselae*, for which the retention time was shorter than that seen for *E*. *coli*. The resistance profiles of bacteria carried by flies often share genotypes with bacteria carried by humans and animals when the habitats of the humans and/or animals overlap with those of the vector [9], suggesting that the bacteria themselves persist in flies. The risk of transmission likely is highest for enteric bacteria, which are shed in high concentrations in human, animals, and fly excrement and are readily ingested by flies. In a previous laboratory study, the abundance (in the fly midgut) of the human opportunistic pathogen *Aeromonas hydrophila*, a bacterium of aquatic origin, was shown to decrease at 24 h post-ingestion, presumably reflecting lysis of these cells within the insect intestine [19]. In contrast to that report, the present study monitored a plasmid, not living bacteria. If the present study instead had quantified the abundance of colony-forming bacteria, the decreases in number may have resembled those seen for *A*. *hydrophila*. Considered together, the results of past reports and of the present study suggest that marine bacteria are capable of spreading an ARG-coding plasmid via insect vectors. The plasmid is expected to be stably retained in the house fly intestine, even if the host bacteria are lysed or in a vegetative state, which may permit the conversion of endogenous intestinal bacteria to ARB. When both the donor and recipient bacteria are actively growing, conjugation between marine bacteria and enteric bacteria has been estimated to occur at rates ranging from 10^−7^ to 10^−3^ [21, 27].

Pathogenic bacteria can be transferred from flies to the environment by mechanical dislodgment from the exoskeleton or via fecal deposition and regurgitation [28]. Among these processes, regurgitation is known to occur significantly more frequently than defecation [19]. The results of the present work showed that excrement (collectively including feces and regurgitated material) contains plasmid even at 7 days post-ingestion, suggesting that the plasmid may persist in the fly digestive tract. The pAQU1 might horizontally transfer from marine bacteria to endogenous intestinal bacteria in flies.

ARGs have been suggested to be genetic pollutants based on evidence from fresh water [29] and soil [30] environments. ARGs also have been isolated from cultured fish and the seawater environment [31]. The present study suggested that the house flies are potential vectors for conveying ARGs from marine settings and materials to human life. When *E. coli* is ingested by adult flies, ARG carriage is maintained throughout the life cycle [32]. ARB have been detected in eggs, larvae, pupae, and the subsequent generation of imagoes. Additionally, if such larvae are fed to chickens, ARB are detected in the chicken intestine for at least 46 days [32]. A separate study showed that flies and cockroaches can harbor multidrug-resistant bacteria and therefore may play a role in the transmission of ARB via pre- and post-harvest food [5]. Thus, all insect-mediated connections between agricultural environments and human life should be managed to decrease health risks, both to animals and humans. Our present study indicated that a plasmid, whether conveyed by an enterobacterium or by a marine bacterium, is retained for multiple hours in the house fly, a time interval sufficient for these flies to move into other environments. Again, these results suggest that the house fly is capable of transmitting ARGs from the marine environment to humans through fish and fishery products. We propose that insects should be considered important carriers and reservoirs of ARGs among human, animal, and water environments and that the One Health concept should be expanded to include the marine environment.

### Intestinal Microflora

**Fig. 2:**
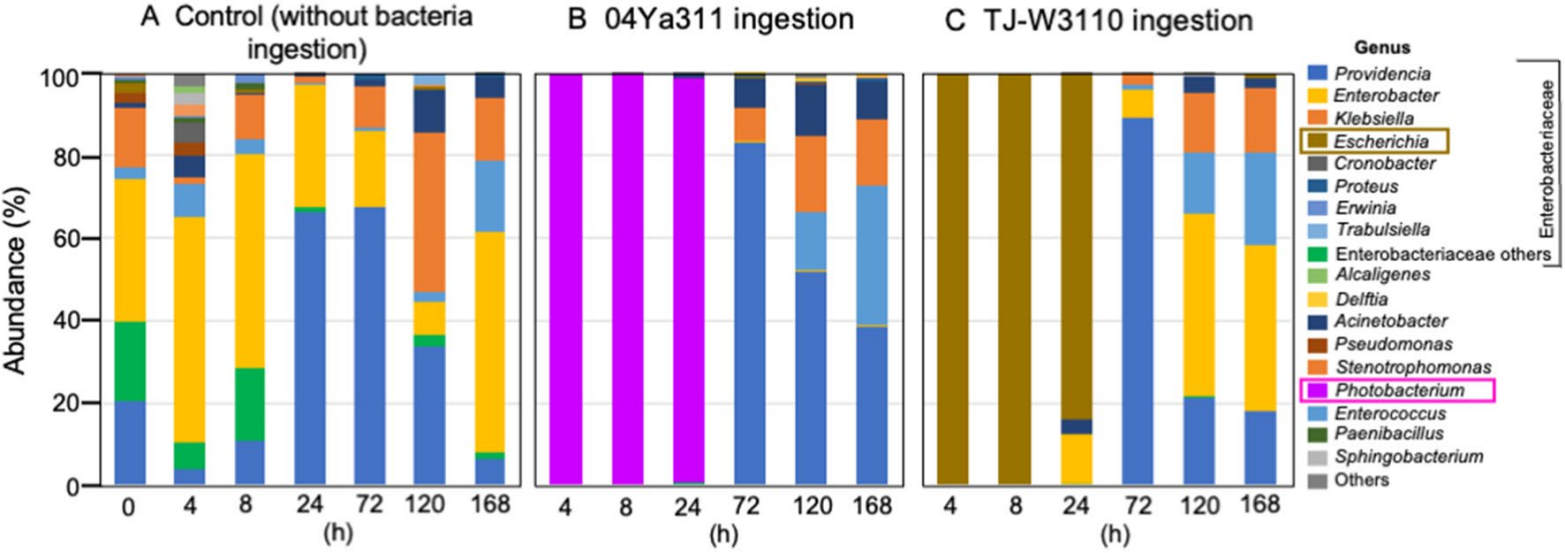
Time course of changes in the intestinal microflora in the house fly following 4 h of feeding on skim milk supplemented with vehicle (PBS) or bacteria. Colors and genera are defined to the right of the plots

As shown in Fig. 2A, Enterobacteriaceae were abundant in the intestine of the control group throughout the experimental period, during which a succession of species (at the genus level) was observed. Specifically, *Enterobacter* was abundant for 8 days, while the proportion of *Providencia* was elevated from 24 to 120 h, and that of *Klebsiella* was increased at 120 and 168 h. *Escherichia* was a minor component of the intestinal microflora throughout the study period. In the 04Ya311-fed flies (Fig. 2B), *Photobacterium* dominated for the first 24 h, progressively falling thereafter before apparently disappearing by 72 h. *Providencia* dominated at 72 h, after which *Erwinia* and *Klebsiella* became abundant. In the TJ-W3110-fed flies (Fig. 2C), *Escherichia* was abundant for the first 24 h, indicating that the ingested bacteria remained abundant for the first day, similar to the results seen in the 04Ya311-fed flies. *Providencia* dominated at 72 h, again similar to the pattern in the 04Ya311-fed flies. The peak abundance of *Providencia* at 72 h was shared among all experimental groups, with the bacterial profile subsequently progressing to distinct genera of Enterobacteriaceae in the different groups. Although TJ-W3110 is an *E*. *coli* isolate, microbes of this genus did not remain abundant after 72 h, even in the flies maintained on TJ-W3110. Other work on ARB carriage in insect vectors has shown that such ARB are primarily enteric bacteria corresponding to microbes from animal farms, wastewater treatment plants, and restaurants [5]. The present study also showed that Enterobacteriaceae are abundant in the typical intestinal microflora of (laboratory-maintained) house flies. Experimentally ingested bacteria replaced the endogenous microflora immediately following feeding; after 72 h, however, the intestinal microflora recovered to represent the usual microflora, consisting largely of Enterobacteriaceae of diverse genera.

**Fig. 3:**
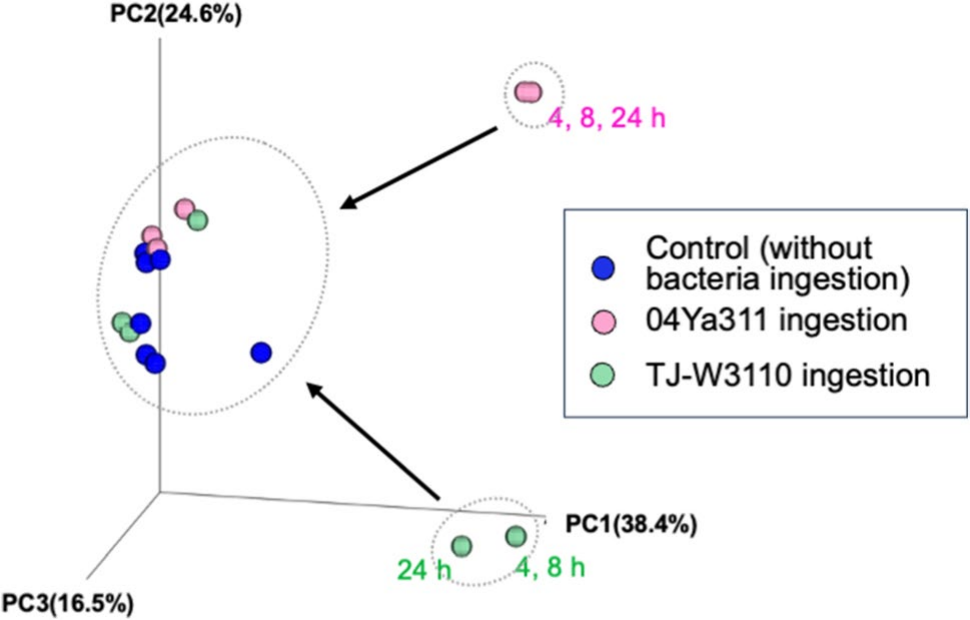
PCoA analysis of the *traI* profile of each specimen

A similar study in the black soldier fly showed that *Ignatzschineria* initially dominates the intestinal microflora of this species, with *Enterococcus* subsequently increasing in abundance over time, especially in the presence of oxytetracycline (OTC). The abundance of *Providencia* was also initially high in that study but decreased with OTC concentration and time of exposure. However, the abundances of *Morganella*, unclassified Enterobacteriaceae, and *Actinomyces* tended to increase over time; such increases correlated with the concentration of OTC [33]. We expect that the presence of antibiotics will select for ARB and a distinct profile of microflora. The recovery of the intestinal microflora in our study is illustrated in Fig. 3. Microflora at 4, 8, and 24 h of the bacteria-fed groups formed distinct clusters, which (over time) then converged with cluster seen in the control group. We expect that the intestinal microflora would be stable in the absence of selective pressure. Drastic changes appeared to be the result of ingestion of large quantities of specific strains of bacteria.

**Fig. 4:**
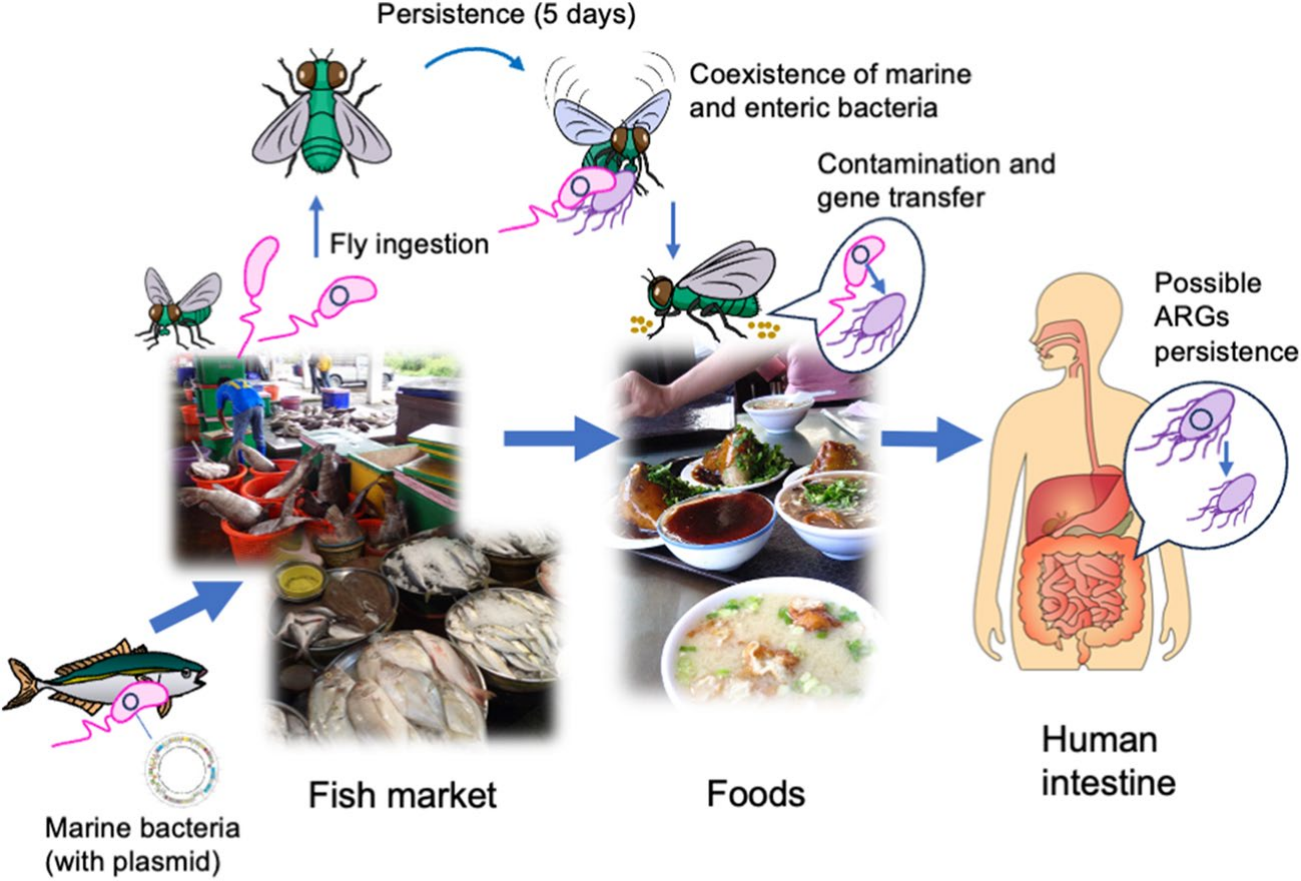
Graphic summary of transmission of marine plasmid to human environment via house fly over time. Possible horizontal gene transfer between marine and enteric bacteria in flies and potential risk to humans by ARGs persistence are included

The marine bacteria (04Ya311) were not detectable at 72 h in flies, whereas the plasmid was detected at 120 h in flies and 168 h in excrement. Since the pAQU1 can transfer to *E*. *coli* [21, 25, 27], it is suggested that the plasmid could be transferred to intestinal bacteria of flies. The enteric bacteria of flies are shared with humans, which suggests the transfer and persistence of marine-derived plasmid (ARGs) to human enteric bacteria. This possibly becomes a risk to humans as shown in Fig. 4. Although marine-derived plasmids have never been proved from insect in actual condition survey at this moment, present experimental study makes an avenue to future ecological and monitoring studies of ARGs. These approaches should contribute to the broader understanding of the antibiotic resistance issues from One Health viewpoint.

## Conclusion

Plasmid pAQU1 was monitored for 7 days in house flies subjected to 4-h access (the pulse) to a plasmid-bearing marine bacterium (Strain 04Ya311) or a plasmid-bearing *E*. *coli* (Strain TJ-W3110), followed by access to skim milk neat (the chase). Notably, pAQU1 was retained in the intestinal content for a longer period in TJ-W3110-fed flies than in 04Ya311-fed flies. However, even with the marine bacteria, the plasmid was still detected for at least 5 days following ingestion of 04Ya311. This interval would be sufficient for transmission to the human environment. Therefore, we propose that the house fly is capable of serving as a vector for the transfer of marine-derived ARGs to humans. Transmission process of ARGs from fish to foods via house fly shown in Fig. 4 includes possible HGT and persistence in humans, which is an ARGs risk from fishery products and environment.

### Supplementary Information

Below is the link to the electronic supplementary material.

Supplementary file1 (TIFF 4234 KB)

## Data Availability

All data analyzed are provided in the manuscript and supplemental file. The raw datasets generated and analyzed during the study are available from the corresponding author upon reasonable request.
