# Supplementary figures and images for: Persistence of Marine Bacterial Plasmid in the House Fly (Musca domestica): Marine-Derived Antimicrobial Resistance Genes Have a Chance of Invading the Human Environment
Source: Microb Ecol. 2024 Jan 8;87(1):30. doi: 10.1007/s00248-023-02341-4 (PMC10774151; doi:10.1007/s00248-023-02341-4)

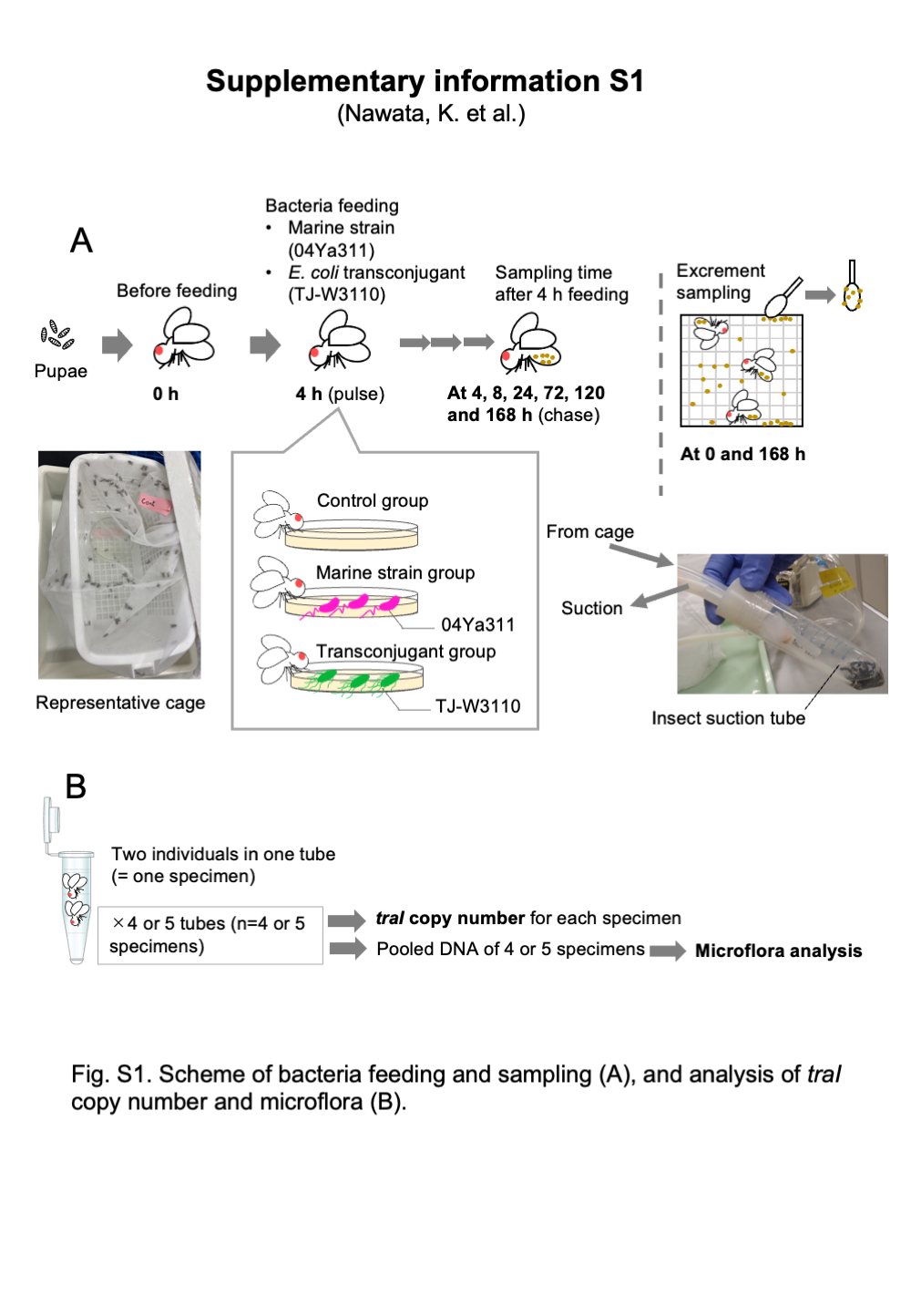

Supplement: Supplementary file 1 — Supplementary file1 (TIFF 4234 KB) [file 248_2023_2341_MOESM1_ESM.tiff]
